# Analysis of Blood Donor Deferrals: Experience From a Newly Established Blood Centre in a Rural Medical College Hospital in a Tribal Area

**DOI:** 10.7759/cureus.81679

**Published:** 2025-04-03

**Authors:** Manoj K Minj, Minal Wasnik, Saurabh Lahare, Kiranlata Bhagat

**Affiliations:** 1 Pathology, Late Shri Lakhiram Agrawal Memorial Medical College, Raigarh, Raigarh, IND; 2 Transfusion Medicine and Blood Bank, All India Institute of Medical Sciences, Raipur, Raipur, IND; 3 Transfusion Medicine, All India Institute of Medical Sciences, Patna, Patna, IND

**Keywords:** blood donation, deferral, hepatitis b surface antigen (hbsag), low hb, permanent, temporary

## Abstract

Introduction

Blood transfusion services (BTS) are an integral part of healthcare. In rural areas, the status of BTS has not been properly studied. We conducted this retrospective study to analyse the donor deferral pattern and understand the reasons for deferral in a newly established blood centre at a rural medical college.

Methodology

This was a retrospective study, where we analysed blood donor deferral data from January 1, 2024, to December 31, 2024. The donor deferral records of both replacement and voluntary donors, maintained in the blood centre, were analysed.

Results

A total of 4,716 donors were registered. Of these, 322 were deferred due to various reasons, with 70.8% (n = 228) being temporary deferrals and 29.2% (n = 94) being permanent deferrals. Males accounted for 94% (n = 303) and females for 6% (n = 19) of the deferrals. Low haemoglobin (Hb) was the most common reason for temporary deferrals, while hepatitis B surface antigen (HBsAg) and syphilis reactivity were the most common reasons for permanent deferral.

Conclusion

This retrospective analysis of donor deferral patterns in a rural hospital showed low Hb, alcohol intake, and HBsAg reactivity as some common reasons for deferrals. As the region is endemic for sickle cell disease, we recommend the inclusion of sickle cell testing in donor screening, as well as efforts to improve the overall health of the rural population.

## Introduction

Blood transfusion services (BTS) are a crucial part of healthcare services, and it is their responsibility to ensure a continuous blood supply by mobilizing the community toward regular, non-remunerated blood donation. A blood centre plays an important role in providing safe blood to patients in need. The safety of blood begins with donor selection and includes several other processes, like testing of donated units, optimum storage, as well as appropriate transfusion practices. Appropriate donor selection is still emphasized, even in the era of sensitive infection testing technologies [1]. A healthy donor is selected based on guidelines provided by the Directorate General of Health Services, Ministry of Health and Family Welfare (MoHFW), Government of India (GOI), and the Drug and Cosmetic Rules (Second Amendment) 2020, dated March 11, 2020. Based on these guidelines, a donor can be rejected from donating blood, which is known as a deferred donor [2]. Blood donor deferral, whether temporary or permanent, is an important step in improving blood safety. Understanding the deferral pattern by analysing the causes and mitigating them, wherever possible, is essential for improving donations.

BTS in rural parts of our country are not well-developed and poorly regulated. The donor screening guidelines, as well as deferral criteria, are not stringently followed. Our blood centre was established in a rural medical college situated in the tribal region of Chhattisgarh state to improve the BTS in the region. The aim of our study was to evaluate and analyse the patterns and causes of blood donor deferrals in a newly established blood centre within a rural medical college hospital. We intended to understand the reasons for blood donor deferrals so that strategies can be implemented to improve donor recruitment and retention [3].

## Materials and methods

After obtaining appropriate approval from the Institutional Ethics Committee (S. No./Med./Ethics Commi./2025/36), this retrospective analysis was performed at the newly established blood centre of a rural medical college hospital in Raigarh, Chhattisgarh. The pre-donation blood donor counselling was performed by a trained counsellor at our blood centre. Data was collected and analysed from donor registration and donor deferral records/registers maintained by the donor counsellor at the blood centre from January 1, 2024, to December 31, 2024.

The donor registration register, along with donor deferral records/registers, were the main source of data regarding total donor registration and deferral pattern, respectively. Inclusion criteria included in-hospital and outdoor blood donation camps, and both voluntary as well as replacement donors. Donors were screened based on criteria laid down by the Directorate General of Health Services, MoHFW (GOI), as per the Drug and Cosmetic Rules (Second Amendment) 2020, dated March 11, 2020, for blood donor acceptance and deferral [4].

Haemoglobin (Hb) estimation was done by the specific gravity method, using copper sulphate solution as departmental standard operating procedures based on procedures described elsewhere [5]. Donors having weight ≥45 kg and Hb ≥12.5 g/dL were eligible for donation. Donors not fulfilling the acceptance criteria were deferred and excluded from donations.

Temporarily deferred donors were deferred for a specific time period, after which they could again become eligible donors; whereas permanently deferred donors were deferred for a lifetime and could never donate blood. The donor registration and deferral data were collected and organized in Microsoft Excel (Microsoft® Corp., Redmond, WA, USA) for further analysis. Descriptive statistics were used to summarize the demographics of the blood donors, including deferral rates and the frequency of temporary and permanent deferrals. Additionally, a Chi-square test was performed to study any gender association with the deferral pattern.

## Results

A total of 4716 potential donors were screened during the study period from January to December 2024. Of these, 4483 donors donated blood, and 322 donors were deferred (deferral rate: 7.2%) for various reasons during this period. Among the 4483 blood donors, 1822 were voluntary and 2661 were replacement donors. Among the deferred donors, 70.8% (n = 228) were temporarily deferred, and 29.2% (n = 94) were permanently deferred. The males accounted for 94% (n = 303) and females for 6% (n = 19), as seen in Table 1. There was a statistically significant difference in the deferral pattern between males and females (p < 0.05). The maximum deferrals occurred in the age group of 35-40 years.

**Table 1:** Gender-wise frequency of temporary and permanent deferrals.

Gender	No. of deferrals (%)	Temporary deferral	Permanent deferral
Male	303 (94%)	209	94
Female	19 (6%)	19	0
Total	322 (100%)	228	94

The commonest reason for temporary deferral was low Hb, i.e., Hb <12.5 gm% (n = 80), with 61 males and 19 females, followed by alcohol intake (n = 74). The temporary deferral data from January to December 2024 can be seen in Table 2.

**Table 2:** Month-wise data of reasons of temporary deferral. Hb: haemoglobin

SN	Month	Low Hb	Underweight	Medications	Alcohol
1.	January	03	-	07	05
2.	February	03	01	05	07
3.	March	06	-	-	07
4.	April	05	-	02	09
5.	May	12	03	05	15
6.	June	05	02	06	07
7.	July	12	-	08	04
8.	August	12	-	10	05
9.	September	06	02	08	04
10.	October	01	-	04	01
11.	November	03	02	02	03
12.	December	12	02	05	07
	Total	80	12	62	74

The commonest reason for permanent deferral was hepatitis B virus (HBV; n = 26) and syphilis (n = 26) reactivity in transfusion-transmitted infection (TTI) testing. In nine donors, we found multiple TTI reactivity. All the donors showing TTI reactivity were males; however, the gender difference was statistically insignificant (p > 0.05). Five donors were also permanently deferred due to high-risk behaviour.

## Discussion

Deferring unfit donors is an important aspect of the blood donation process, particularly in rural blood centres, where it is vital to have a safe and sufficient supply of blood. Deferrals can result in a critical shortage of blood units, especially in rural areas where healthcare resources are already stretched thin. Our blood centre is newly established in a rural medical college in Chhattisgarh state, which has a large tribal population and limited awareness about blood donation. This region is endemic for haemoglobinopathies like sickle cell anaemia [6,7]. Therefore, it is crucial to understand the donor deferral pattern in such regions to improve blood donation strategies and ultimately improve public health.

Since the blood centre became functional, 4716 potential donors were screened, out of which 4483 were accepted and 322 donors were deferred. The deferral rate was 7.2%. The donor deferral rate varies from region to region. Malhotra and Negi found a deferral rate of 17% in North India [1], whereas in South India, it was observed to be 5.85% [3]. Shah et al. found a deferral rate as high as 33% in Western India [8]. The deferral rate may also vary from centre to centre within a region. A study in a teaching hospital in an urban area of Chhattisgarh by Kujur et al. showed a deferral rate of 3.8% [9], indicating variation in the donor population. Out of the total deferred, males were 94% and females were 6%. Prajapati and Parmar observed a deferral rate of 83.5% in males and 16.5% in females in a rural hospital in the western region of India [2]. Several other studies also show a similar pattern [10-12]. The low deferral rate in females is due to low female participation in the donation process, owing to various factors like rural areas, lack of awareness, fear of anaemia/weakness, fear of needles, etc.

Among the 322 deferrals, 228 (70.8%) were temporary and 94 (29.2%) were permanent deferrals, which is comparable to other studies [2,9]. The commonest reason for temporary deferral was low Hb in both males and females. In rural areas, poor nutrition due to low socio-economic status can lead to anaemia. Besides this, it could be due to sickle cell disease, as the region is endemic. We suggest that screening tests for sickle cell disease be included as part of blood donor screening. The second most common reason for temporary deferral was alcohol intake within the last 24 hours. A system should be established to follow up with donors who are temporarily deferred, so that they can be included in the donor pool again.

The common reasons for permanent deferrals included HBV and syphilis reactivity in 26 cases each, which were tested by ELISA (Enzyme-Linked Immunosorbent Assay). The reactive donors should receive adequate post-donation counselling and referrals for further management. A stringent donor screening should be conducted to elicit a history of tattooing and piercing, as these are prevalent practices in rural areas.

Deferral of donors is an issue that must be given particular attention by blood centres, especially those located in rural areas. A low literacy rate and low socioeconomic conditions in such areas make it necessary to develop specific strategies for motivating and recruiting donors. Zou et al. found a negative impact of temporary deferral on the donor return rate [13]. It is important to provide such donors with proper counselling and management to enhance the effectiveness of the donor program. Overall, most deferrals in our study were temporary, which can be retrieved for donations through adequate follow-up [14]. Health authorities should establish policies aimed at improving the overall health of the community in this region. Policies like sickle cell screening on a mass level, population-based genetic testing and counselling, improving literacy rates, nutrition supplementation, and donor education programs promoting healthy lifestyles will make an impact on improving blood donations.

Although our study provided an overview of the deferral pattern in our region, it is not free of limitations. We had lower female participation and could not perform sickle cell screening due to the unavailability of the testing facility at our blood centre. Additionally, we did not study the effect of socioeconomic factors, cultural and religious beliefs, and ethnicity (tribal/non-tribal) on donor deferral patterns. These could have helped further analyse their role in blood donor recruitment.

## Conclusions

The assessment of deferral reasons and deferral rates, with a distinction between temporary and permanent deferrals, can significantly aid medical professionals involved in blood collection by refining their donor screening process. It is imperative that all potential donors who are temporarily deferred receive adequate information, counselling, and education regarding the reasons for their deferrals. Enhancing public awareness about blood donation - especially among females, the reasons for deferral, and a focus on improving overall health - may effectively reduce deferral rates and promote retention of prospective donors. In rural areas, local newspapers, radio stations, and talks/short movies during local festivals, fairs, and markets may be used to spread awareness. Additional research, including a larger tribal population, needs to be carried out to understand the deficiencies and conditions of BTS in the state. It will help improve blood donations and ultimately the overall healthcare services of the state.
